# Body Mass Index and Dental Caries, a Five-Year Follow-Up Study in Mexican Children

**DOI:** 10.3390/ijerph18147417

**Published:** 2021-07-11

**Authors:** Leonor Sánchez-Pérez, Laura Patricia Sáenz-Martínez, Nelly Molina-Frechero, María Esther Irigoyen-Camacho, Marco Zepeda-Zepeda, Enrique Acosta-Gío

**Affiliations:** 1 Departamento de Atención a la Salud, Universidad Autónoma Metropolitana, Unidad Xochimilco, Ciudad de México 04960, Mexico; lpsaenz@correo.xoc.uam.mx (L.P.S.-M.); nmolina@correo.xoc.uam.mx (N.M.-F.); meirigo@correo.xoc.uam.mx (M.E.I.-C.); mzepeda@correo.xoc.uam.mx (M.Z.-Z.); 2 Laboratorio de Microbiología, Posgrado de la Facultad de Odontología, Universidad Nacional Autónoma de México, Ciudad de México 04360, Mexico; acostag@unam.mx

**Keywords:** body mass index (BMI), dental caries, caries incidence, follow-up study, Mexican children

## Abstract

There are conflicting reports on a possible association between body mass index (BMI) and caries. Given the ongoing worldwide increase in obesity, we undertook a 5-year follow-up study on 201 Mexican schoolchildren to analyse their BMI and dental caries experience. The children’s weight and height were recorded, and their BMI was calculated using the WHO tables. Decayed, missing, and filled surfaces in both dentitions (dmf/DMFS) were assessed annually according to WHO criteria by two calibrated researchers (Kappa value 0.92 *p* < 0.001). The means, standard deviation, an ANOVA, and Student’s *t*-test were calculated to analyse the relationship between the variables. At baseline, the children had an average of 6.5 ± 0.5 years, a BMI of 17.2 ± 3.1 (CI_95%_ 16.8–17.6). Their weight’s classifications were 61% normal, 19% obese, 17% overweight, and 3% showed thinness. At the end of the study, their BMI were 20.6 ± 4.4 (CI_95%_ 19.8–21.5), 53% normal, 15% obese, 30% overweight, and 2% thin. The children’s dmfs decreased from 5.8 ± 9.2 to 1.8 ± 3.4 and the DMFS increased from 0.07 ± 05 to 1.4 ± 2.3. In this population based on a 5-year follow-up, caries prevalence and incidence were not significantly associated with the BMI. However, schoolchildren with malnutrition had the highest caries indexes.

## 1. Introduction

Internationally, dental caries remains a public health problem; it is considered to be the most common chronic disease during childhood and its effects are cumulative in the permanent dentition [1]. An unhealthy diet, high in carbohydrates, has been associated with increased caries rates [2]. In Mexico, more than 70% of the child population is affected by this disease [3].

Childhood obesity (O) is a problem of alarming proportions worldwide, which is higher in underdeveloped countries [4]. Children who are overweight (OW) or O present a higher risk of developing systemic diseases such as metabolic syndrome, diabetes, fatty liver and muscle disorders among others [4]. Some European countries have reported data on child OW and O between 30 and 52% [5]. Among schoolchildren in Mexico, in 2016 the prevalence of OW was 17.9% and of O was 15.3% [6].

The environments in which children are growing up are conducive to weight gain and O. Globalization and urbanization are contributing to the increased exposure of unhealthy (obesogenic) environments in high-, middle-, and low-income countries, and in all socioeconomic groups [7].

The marketing of energy drinks and nutrient-poor foods has been identified as one of the main reasons for the increase in the number of OW and O children. The impact of these factors on dental caries is under discussion at national and international levels [8,9].

Published reviews have shown contradictory evidence, with reports of both positive and negative relationships between caries and obesity [9,10,11,12,13,14,15]. It has been suggested that longitudinal studies are needed to understand the possible interaction between obesity and caries [13].

The aim of this five-year follow-up study was to analyse the body mass index (BMI) and dental caries in a group of Mexican schoolchildren.

## 2. Materials and Methods

### 2.1. Ethical Considerations

This observational follow-up survey adhered to relevant guidelines for appropriate ethical research design and the relevant standards to ensure the protection of human subjects. The protocol was reviewed and approved by the Research Commission and Ethical Committee at the Autonomous Metropolitan University—Xochimilco (IRB: 14/10, 10/17, CE.002.17). The parents of all the children enrolled in the first year at one elementary school were invited to allow their children’s participation. Before taking any measurements, informed consent was obtained in writing from the parents, allowing 280 of 300 children to participate. No data or samples were collected from 20 children whose parents declined their children’s participation. Parents were informed periodically of the results of their participating children’s examination and advised about the institutions that could provide dental and medical services for their community. All the children received oral health education sessions and new toothbrushes were distributed free of charge every year.

### 2.2. Participants 

In 2011, we initiated this 5-year follow-up study in a public (state funded) school in southern Mexico City. The school is located in a middle-income area of the city. Table salt available in Mexico City is all fluoridated. In this area, the fluoride content in water was <0.247 ppm. Based on their places of residence, the children were classified into one of two socioeconomic (SE) categories: middle and middle–low. In accordance with the classification developed by the Instituto Nacional de Estadística, Geografía e Informática (Mexico’s National Institute of Geography and Informatics), using security, education, employment, and quality of housing as indicators for the neighbourhood level.

Of 280 6-year-old children that participated at baseline, 79 children dropped out from the study (28% drop-out rate). The main reasons for their exclusion were changing schools, not being available, an unknown reason or the child not attending two anthropometric measurements. Student’s *t*-test comparison of the drop-outs (51% boys) and the children remaining in the follow up showed that the caries average and BMI value were similar: *p* = 0.145, *p* = 0.1509, respectively. No information about drop-outs is presented in the results. The final sample consisted of 201 schoolchildren.

### 2.3. Survey Instrument

Parents completed questionnaires about oral hygiene habits and the consumption of snacks and soft drinks, at baseline and at yearly intervals. The questionnaire information collected at baseline was used in the analysis.

### 2.4. Dental Examination

Two calibrated examiners (Kappa = 0.95) performed the oral examination for caries prevalence at baseline using a natural light source, a mirror, and a periodontal probe. The caries prevalence was scored as the sum of decayed, missing, and filled deciduous surfaces (dmfs) and decayed, missing, and filled permanent surfaces (DMFS), according to the World Health Organization criteria [16]. 

To determine the caries increment, the oral examination was repeated every year by the same examiners. To avoid bias, the schoolchildren were evaluated without access to their caries record. For each child, the caries increment was calculated by subtracting the baseline dmfs or DMFS score from the new dmfs or DMFS score. 

### 2.5. Anthropometric Measurements

The children’s weight and height were also measured biannually, and the BMI was calculated, given it is the most widely used index in dentistry. A standardized protocol was followed to perform the anthropometric measurements [17]. These measurements were carried out by one nutritionist, who was previously trained in anthropometric measurements in children. The intraclass correlation coefficient (ICC) was 0.91–0.96 for height and 0.90–0.95 for weight. The same nutritionist performed all anthropometric data in each measurement of the study. The participants were weighed in light clothing without shoes. Height was measured using a stadiometer (Seca, Hamburg, Germany), and for weight, a standard clinical scale (Seca, Hamburg, Germany).

Using age and sex specific criteria, BMI for age growth chart standards (WHO) [18] were applied to obtain the percentile values and were categorized as: underweight < 5% normal > 5% to <85% overweight 85% to <95% and obese ≥ 95%.

### 2.6. Statistical Analysis

The mean and standard deviations (±) and proportions for the main variables by year of follow up were calculated. The average BMI was computed, considering the trajectory of the children during the follow-up period. ANOVA was used to estimate differences in the means of the selected variables at the beginning of the study. Analysis of Variance (ANOVA) or t Student’s *t*-test was applied to compare the mean dmft/DMFT by BMI and sex. The data was analysed using JMP 11 software (SAS Institute Inc., Cary, NC, USA). A *p*-value of less than 0.05 was considered statistically significant.

## 3. Results

Of the 201 schoolchildren, 52% (*n* = 104) were girls. The average age at the start of this five-year study was 6.5 ± 0.5. At baseline, the schoolchildren on average brushed twice a day, had four meals a day and drank two soft drinks a day (600 mL, between 21 to 70 glucose mg/mL), with no significant differences between sex (*p* = 0.1136; *p* = 0.1505; *p* = 0.23023, respectively).

The caries prevalence was 41% at the beginning of the study and 40% at the end. The dmfs decreased 6.3 ± 11.0 to 2.1 ± 3.9 and the DMFS increased 0.03 ± 02 to 1.1 ± 1.9. No significant differences were observed by sex in dmfs, DMFS or BMI. In the follow-up study, the BMI of the group increased with age; this and their confidence intervals are shown in Table 1.

During follow up, the number of children with a normal BMI decreased from 123 at baseline to 107 at the end of the study. The number of OW children increased, and the number of O children decreased.

OW increased with age, and O was the variable with more variations. When OW and O were grouped together, the research found a prevalence of 36%, which increased to 45% at the end of the follow up (Table 2). The average BMI in the first and last measurements were: for thin children: initial 13.0 ± 0.7 final 14.1 ± 0.3; normal: 15.5 ± 0.9 initial and 17.5 ± 1.6 final; in OW children: 18.0 ± 0.9 initial and 21.7 ± 1.6 final, and in O children: initial 22.4 ± 3.0 and final 27.0 ± 2.8 (data not shown).

Of 123 children with a normal BMI at the beginning, 94 retained that category, 2 changed to thinness, 26 became OW and 1 became O. Of the OW children, 23 maintained that category, 8 became normal and 3 changed to O. Of the 39 schoolchildren initially O, at the end of the study, 29 maintained that category, 12 became OW and 1 became normal.

When analysing initial and final dmfs/DMFS and caries incidence rates versus BMI, thin schoolchildren presented the highest caries index and incidence. However, only in the final dmfs index was a significant difference established, *p* = 0.0001. These 3 schoolchildren had on average 4.3 ± 5.1 deciduous teeth present (between 4 and 10 teeth).

The incidence of dental caries in the permanent dentition and the total incidence were equal among the four groups (Table 3). In addition, the incidence of dental caries by sex was not different *p* = 0.9628 (data not shown).

Grouping low and normal, or OW and O, there were no significant differences between BMI and the caries incidence. (Table 4).

## 4. Discussion

The results of this 5-year follow-up study suggest that there is no association between caries experience and different BMI categories among six- to twelve-year-old schoolchildren.

The initial and final caries prevalence were 41% and 40%, respectively; the mean dmf/DMFS score and the overall dental caries were 1.8 and 1.4, respectively, and the final total caries incidence was 5.1 surfaces. The caries prevalence in this study was similar to that reported in this decade by the Third National Caries and Fluorosis Survey [3]. The prevalence of caries was higher in primary teeth; our results suggest that only one association was found between dmfs and thinness (*n* = 3), and due to the initial high dmfs and the persistence of the deciduous teeth, no other associations were found. 

In the last few decades, the prevalence of OW and O has increased worldwide, with the number of children affected ranging from 31 to 41 million. The number in developing countries has doubled from 7.5 to 15.5 million. Childhood obesity is associated with a wide range of serious health complications and an increased risk of contracting diseases prematurely, including diabetes and heart disease [19].

The Health and Nutrition Survey in Mexico reported a combined prevalence of OW-O in the school population between 5 and 11 years old of 37.9%. The overall prevalence of OW was 18.4% and O 19.5%. At the age of twelve, 36.1% of the population was reported to be in the OW-O category [20]. In this investigation, 45% of the children studied were in this category, which may be due to the size of the sample or to the fact that some children are in development.

Diet is of utmost importance in developing OW-O and dental caries. Some authors have described that the risk factors to develop this binomial include poor dietary habits, the frequency and consumption of carbohydrates, juices, sodas, and high-calorie diets [20,21]. As children develop, they become more independent in their choice of food, allowing this binomial of OW-O and dental caries to increase [13,22].

We observed that 53% of the children maintained their initial BMI category; of which, 37% (*n* = 75) maintained a normal BMI during the five years of follow up, 4% (*n* = 8) maintained their BMI in OW and 12% (*n* = 24) were O in all measurements. Of the children who recorded thinness at the end of the study, two of them started with normal weight and from the fourth measurement they were recorded as thin. The other 47% oscillated in the follow up.

Systematic reviews of the literature suggest that there is no consensus on the relationship between BMI and dental caries; some studies show a relationship between both variables, others suggest an inverse relationship, while others express that such a relationship cannot be demonstrated [9,11,12,13,14]. However, there are studies that report finding more cavities among children with OW and O in both dentitions in high-income countries, but not in low- and middle-income countries [23,24]. 

With this background, making comparisons of the data obtained is a complex task, since the reports depend on the age of the schoolchildren and the severity of the dental caries in the country, as well as on intangible situations such as the consumption of refined carbohydrates, starches and fats, which can increase the risk of obesity and, depending on the type and frequency of food consumption, it may also increase the risk of dental caries [6,13,25].

The prevalence of OW-O has been reported to be greater than 20%, [5,21,23,24,25,26] and higher in boys than in girls [25]. Our findings suggest that this percentage varies depending on the age at which the information is obtained; OW started at 6–7 years-old for 16.9% and almost doubled at 12 years old for 30.3%, while OW had higher values than reported, and started at 19.4%, fluctuated during the 5 years of follow up and was 14.9% at the end of the study (11–12 years-old). In relation to sex, as opposed to other studies, we found no differences between sex and nutritional status in any of the observations [25].

The prevalence of caries in our study was 40%, similar to that reported in other countries such as China (41%) [25] and India (43%) [27], and lower than in Germany (50%), [23] or Brazil (59%), [26]. Of these studies, three were cross-sectional [23,25,27] in schoolchildren between 6 and 12/18 years-old, and one was a 2.5-year follow-up study of 12-year-olds [26] Cheng et al. in China [25] reported higher caries rates in the primary dentition, similar to this study, and Reddy et al. in India [27] found that underweight children had more caries.

Concerning the association between BMI and dental caries, Willerhausen et al. in Germany and Reddy et al. in India reported an association and Cheng et al. did not [23,25,27]. The study by Lock et al. in Brazil [25] reported that after a 2.5-year follow up, 12-year-old OW/O children have fewer dental caries lesions, a higher DMFS and no association between BMI and caries, [26] similar to our data. On the other hand, concurrent with most literature reviews [1,8,13,14], in this investigation, no association was found between dental caries and BMI.

The limitations of the present study were to establish the criteria for obesity, sample size and losses during follow up. Our results suggested a minimal impact in relation to dental caries, but not in relation to overweight or obesity.

The outlook of overweight and obesity is very complex, it can be suggested that it is determined in part by the increase in the consumption of processed foods with higher amounts of fat, sugars and salt that are consumed due to the working conditions of parents, the tendency to consume fast food, and the important decrease in physical activity [28]. 

The failure to provide early attention to caries lesions in primary and permanent dentition makes it necessary to expand and maintain promotion, prevention, protection, and control actions that allow the government to preserve the oral health of schoolchildren. Likewise, menus should be implemented at school level for children’s break time with healthy food.

We did not find an association between OW-O and dental caries; the current diet is rich in carbohydrates and fats, the origin of a nutritional transition that includes sedentary activities and a high consumption of sugary drinks, which can partially explain the percentages of OW and O. However, one should also consider that the consumption of certain foods causes important changes in the intestinal microbiota that contribute to the development of obesity and insulin resistance, as suggested by some authors [28]. These are elements that, unfortunately, were not considered in this study. 

Dental caries and overweight/obesity share a high carbohydrate intake in the diet. In order to generate strategies to control both public health problems, the possible association between BMI and caries has been studied, with contradictory results, confirming that both conditions are multifactorial.

## 5. Conclusions

In this population, based on a 5-year follow up, the BMI was not associated with dental caries. Overweight and obesity were high in the population studied. A total of 57% of schoolchildren maintained their BMI during the follow up. In 43% of the children, their BMI fluctuated.

## Figures and Tables

**Table 1:** Caries and body mass index (BMI) by sex in the follow-up study.

Age	Year	dmfs		DMFS		BMI		BMI CI_95%_
		Mean ± Sd	*p* Value	Mean ± Sd	*p* Value	Mean ± Sd	*p* Value	
6.5 ± 0.5	Baseline	6.3 ± 11.0	0.4516	0.03 ± 0.2	0.2740	17.2 ± 3.1	0.2571	16.8–17.6
7.5 ± 0.5	1	6.9 ± 10.5	0.3447	0.1 ± 0.4	0.0554	18.1 ± 3.5	0.4334	17.6–18.6
8.6 ± 0.5	2	5.9 ± 7.9	0.4825	0.2 ± 0.5	0.0650	18.9 ± 3.6	0.3274	18.4–19.4
9.6 ± 0.5	3	4.5 ± 6.5	0.2991	0.4 ± 0.9	0.0501	19.5 ± 3.7	0.1866	18.9–20.0
10.6 ± 0.5	4	2.9 ± 5.8	0.2877	0.6 ± 1.3	0.0532	20.2 ± 3.9	0.2014	19.6–20.7
11.6 ± 0.5	5	2.1 ± 3.9	0.2387	1.1 ± 1.9	0.2309	20.6 ± 4.4	0.8438	19.8–21.5

dmfs = decayed, missing and filled deciduous surfaces; DMFS = decayed, missing and filled permanent surfaces; BMI = body mass index; ± = standard deviation; *p* value using Student *t*-test. CI_95%_ = Confidence interval at 95%.

**Table 2:** BMI distribution in the follow-up study.

Year	Thinness	Normal	Overweight	Obesity	% Overweight	% Obesity
**Baseline**	5	123	34	39	16.9	19.4
**1**	3	119	30	49	14.9	24.4
**2**		110	45	46	22.4	22.9
**3**	3	99	55	44	27.4	21.9
**4**	4	101	61	35	30.3	17.4
**5**	3	107	61	30	30.3	14.9

**Table 3:** Initial and final caries index and incidence in the five-year follow up.

BMI	Initial Caries		Final Caries		Incidence		
	dmfs	DMFS	dmfs	DMFS	Deciduous	Permanent	Total/S
Thinness	12.0 ± 14.2	-	12.0 ± 9.3	3.2 ± 3.6	6.2 ± 1.9	3.2 ± 3.6	9.4 ± 11.5
Normal	5.3 ± 8.8	0.1 ± 0.3	1.8 ± 2.9	1.4 ± 2.4	3.7 ± 0.4	1.4 ± 2.4	5.2 ± 5.4
Overweight	5.9 ± 7.7	0.03 ± 0.2	1.2 ± 2.1	0.7 ± 1.3	4.2 ± 0.7	0.8 ± 1.4	4.9 ± 4.9
Obesity	6.5 ± 11.0	0.2 ± 1.0	0.9 ± 2.0	1.3 ± 2.6	3.2 ± 0.7	1.2 ± 2.1	4.4 ± 4.9
Total	5.8 ± 9.2	0.1 ± 0.5	1.8 ± 3.4	1.4 ± 2.3	3.8 ± 4.3	1.3 ± 2.3	5.1 ± 5.4
*p* Value	0.4186	0.4546	0.0001	0.1651	0.4734	0.1394	0.2877

dmfs = decayed, missing and filled deciduous surfaces; DMFS = decayed, missing and filled permanent surfaces; *p* value using one-way ANOVA with Tukey test.

**Table 4:** Distribution according to caries experience and grouped BMI.

BMI	Initial Caries		Final Caries		Incidence		
	dmfs	DMFS	dmfs	DMFS	Deciduous	Permanent	Total/S
Low and Normal (*n* = 128)	5.6 ± 9.0	0.04 ± 0.3	2.1 ± 3.9	1.5 ± 2.5	3.8 ± 4.4	1.5 ± 2.5	5.3 ± 5.7
Overweight and Obesity (*n* = 73)	6.2 ± 9.6	0.1 ± 0.7	1.0 ± 2.1	1.1 ± 2.1	4.2 ± 4.2	1.0 ± 1.8	4.6 ± 4.8
*p* Value	0.6425	0.3783	0.0568	0.2477	0.7620	0.3289	0.4152

dmfs = decayed, missing and filled deciduous surfaces; DMFS = decayed, missing and filled permanent surfaces; *p* value using Student *t*-test.
